# Controlled Mechanical Cracking of Metal Films Deposited on Polydimethylsiloxane (PDMS)

**DOI:** 10.3390/nano6090168

**Published:** 2016-09-09

**Authors:** Andreas Polywka, Luca Stegers, Oliver Krauledat, Thomas Riedl, Timo Jakob, Patrick Görrn

**Affiliations:** 1 Chair of Large Area Optoelectronics, University of Wuppertal, Rainer-Gruenter-Str. 21, Wuppertal 42119, Germany; a.polywka@uni-wuppertal.de (A.P.); luca_stegers@uni-wuppertal.de (L.S.); o.krauledat@gmail.com (O.K.); tjakob@uni-wuppertal.de (T.J.); 2 Institute of Electronic Devices, University of Wuppertal, Rainer-Gruenter-Str. 21, Wuppertal 42119, Germany; t.riedl@uni-wuppertal.de

**Keywords:** cracking, control, stretchable, PDMS, lithography, silver, electroless deposition

## Abstract

Stretchable large area electronics conform to arbitrarily-shaped 3D surfaces and enables comfortable contact to the human skin and other biological tissue. There are approaches allowing for large area thin films to be stretched by tens of percent without cracking. The approach presented here does not prevent cracking, rather it aims to precisely control the crack positions and their orientation. For this purpose, the polydimethylsiloxane (PDMS) is hardened by exposure to ultraviolet radiation (172 nm) through an exposure mask. Only well-defined patterns are kept untreated. With these soft islands cracks at the hardened surface can be controlled in terms of starting position, direction and end position. This approach is first investigated at the hardened PDMS surface itself. It is then applied to conductive silver films deposited from the liquid phase. It is found that statistical (uncontrolled) cracking of the silver films can be avoided at strain below 35%. This enables metal interconnects to be integrated into stretchable networks. The combination of controlled cracks with wrinkling enables interconnects that are stretchable in arbitrary and changing directions. The deposition and patterning does not involve vacuum processing, photolithography, or solvents.

## 1. Introduction

Stretchable electronics is an emerging field of electronics [1,2]. It conforms to curvy surfaces allowing for non-planar optoelectronics [3], tunable optics [4,5,6], stretchable displays [7], solar cells [8], or sensors [9]. Soft biointegrated devices are implanted into biological tissue for much better long-term performance compared to rigid implants [10].

While nanowire and nanoparticle-loaded elastomers enable intrinsically stretchable interconnects [11,12], many electronic applications rely on the low roughness and sheet resistance of classical metal interconnects. Such stretchable metal interconnects can integrate electronic devices into stretchable networks [13]. The goal of stretchable electronics has been to avoid cracking of these metal interconnects. The approach presented here makes use of controlled cracking in order to make the resulting electronic networks more stretchable.

The different methods that have been developed in order to improve stretchability often require patterning [14,15]. For example photolithography on stretchable substrates remains extremely challenging because of the large swelling or shrinkage of elastomers in different solvents [16]. Hence, the realization of solvent-free patterning is highly desirable in the field of stretchable electronics. 

One well-established approach to produce stretchable films even without patterning is wrinkling [17,18,19]. Here, the film is not removed on certain substrate sites. In contrast, during wrinkling only the film surface geometry is influenced. It is compressed by the release of a pre-strain that is applied before film deposition. This way, even large area continuous metal thin films have been demonstrated to become stretchable in arbitrary directions [20]. However, changing the stretching direction causes reorientation of the wrinkles eventually resulting in cracking. Stretching into repeatedly-changing directions can be realized with wrinkled films only, when additional patterning is introduced. Wrinkled networks created by solvent-free patterning enable reliable stretchability in arbitrary and changing direction [21]. 

Still, a few requirements must be fulfilled to use wrinkling in stretchable electronics. Firstly, the fraction of the surface covered by wrinkles, which we term area coverage, is limited, as wrinkles always align with respect to the patterned features [22]. Hence, in order to enable isotropic stretching under changing directions only a limited fraction of the area can be covered by stretchable features. Secondly, the thickness of the wrinkled films or stack of films must not exceed a certain value. Otherwise, the flexibility and, hence, the ability to be locally bent is decreased, which results in cracking. If either the area coverage or thickness of the wrinkled electronics exceed a specific limit, the system will crack under strain.

As cracking destroys existing thin films, this phenomenon is usually considered undesirable. Cracks always appear first in the most brittle areas [23,24,25]. This typically means that the thin films with electronic functionality, i.e., metals, dielectrics, or semiconductors, crack, while the softer environment stays unaffected. However, this also hints to the possibility of controlling cracks by the introduction of surface patterns. While the combination of controlled cracking with lithographic approaches has been suggested for patterning [26,27], it has not yet been investigated whether controlled cracking can totally prevent statistical cracking.

**Figure 1:**
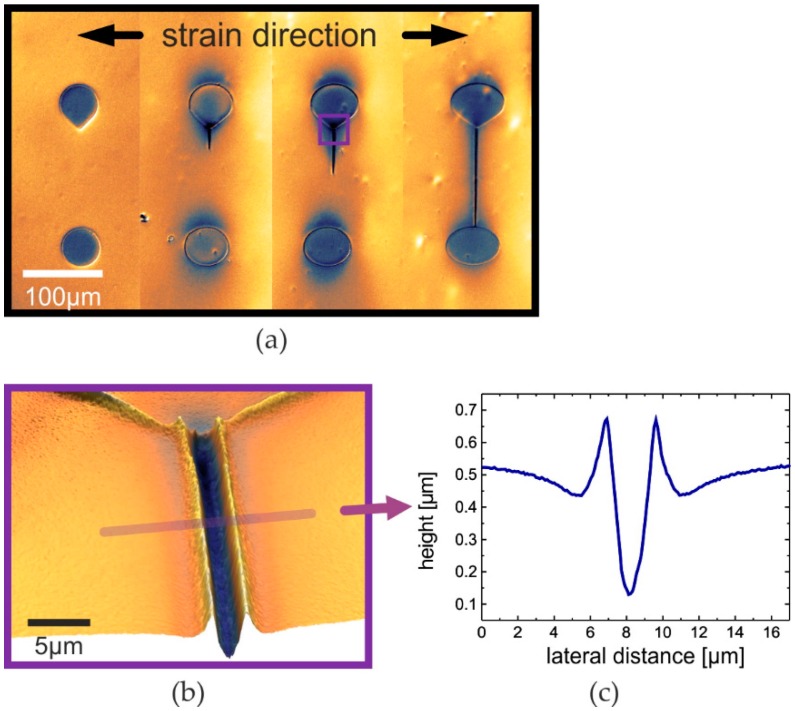
The idea of controlled cracking. Surface profiles from white light interferometry of soft islands on a hardened PDMS surface under various strain; (**a**) increasing strain (left to right) leads to controlled cracking at the tip of the upper island and crack tip propagation towards the bottom island until the crack is fully stabilized (right); and (**b**) a controlled crack with (**c**) the corresponding height profile.

This idea is pursued in this paper. Instead of defining rigid areas within a softer environment capable of releasing mechanical strain, the whole area is hardened. Only very small islands are kept soft, which defines the exact opposite of the rigid island approach, that is well known in stretchable electronics [13]. Accordingly, we term our concept the *soft island approach*. Here, the islands are not the sites to carry the electronic devices but they are a means for controlling the location and direction of cracks. Specifically, a tip attached to one island, which is heading towards a neighboring island, defines the direction of a starting crack. With the substrate stretched perpendicularly to that direction a crack is formed at the soft island’s tip (see Figure 1). While other groups have used large aspect ratio surface patterns like notches to start cracks [26,27,28], the soft islands presented here are the first planar patterns used for that purpose. We have found that the strain needed for that crack formation increases with increasing curvature radius of the island’s tip. The tips prepared here show a curvature radius of around 3 µm.

Due to the local shearing force the crack tip is then propagating away from the island. The crack tip is instable in a way that further mechanical stress leads to its propagation or, in other words, to further growth of the corresponding crack [29]. If the crack is controlled and directed towards a soft island, the crack will finally merge with that island. Afterwards, the crack is stabilized, as both sides are ending in a soft elastomer incapable of further cracking. Nevertheless, such stabilized cracks do open when stretched perpendicular to the crack direction [30]. We will term that phenomenon *crack expansion*. Our observations are, therefore, in good agreement with the theoretical analysis of the local strain distribution in cracked films. This way stabilized cracks enable local tensile strain without destruction of the rest of the film. With the positions of these stabilized cracks being well-controlled, one can remove films or whole devices in the proximity of these positions. This new kind of cracking will, therefore, not lead to destruction of the electronic functionality. On the contrary, it will add a new way to prevent uncontrolled cracking.

## 2. Results

In this paper, we present an application of the described concept that is illustrated by Figure 1 in stretchable metal interconnects. A sapphire mask coated with chromium at the sites defining the soft islands is placed on the PDMS substrate. The substrate is then illuminated with UV (172 nm) radiation through the mask. This way the surface is hardened except for the small island areas where the UV is blocked by the chromium. These island stay elastomeric so they will not crack under mechanical strain. The hardened area on the other hand will crack during mechanical stretching. To investigate cracking different types of samples are prepared. First cracking of the hardened PDMS surface is studied without silver films.

### 2.1. Cracking of the UV Hardened PDMS Surface

The critical strain for cracking of the UV hardened PDMS surface can be tuned with the UV-dose [21]. Without treatment no cracks are observed as the PDMS stays elastomeric. At large doses very brittle silica-like films can be prepared that crack at low strain levels of less than 1%. In that sense the UV dose is a first parameter to control the critical strain for cracking which defines the maximum elongation allowed during fabrication and handling of the substrates. In the following studies a dose of 30 J·cm^−2^ (much lower at the PDMS surface, see Section Materials and Methods) was applied, leading to a critical strain for the onset of uncontrolled cracking of about 18%. This value enables simple handling of the substrates without introducing uncontrolled cracks. At this dose the crack depth saturates at a strain of 25%. We conclude that the saturation crack depth of 950 nm corresponds to the thickness of the hardened PDMS. The overall thickness of the PDMS substrate is about 1 mm.

**Figure 2:**
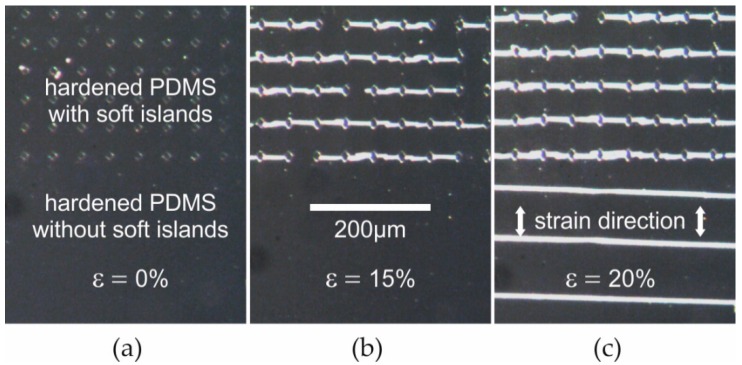
Optical micrographs of PDMS surface treated with UV (30 J·cm^−2^): top area: including untreated soft islands (distance of 45 µm), bottom area: homogeneously-hardened; micrographs taken after application of strain (direction shown in c) of 0% (**a**); 15% (**b**); and 20% (**c**).

Figure 2 shows the crack-control introduced by the soft island patterns. At small strain (Figure 2a) the islands can be seen at the top area of the sample. The sample is now stretched in a vertical direction with the same strain applied to the top area including soft islands and to the homogeneously-hardened bottom layer. At a strain of about 15% (Figure 2b) most of the controlled cracks are fully propagated between two soft islands and have, in this way, been stabilized. At about 18% uncontrolled cracks appear in the homogeneously-hardened bottom region. It is observed that the density of these undesired cracks is lower compared to the controlled ones at the top area (Figure 2c). Additionally, their orientation is slightly different, showing that the external strain was not perfectly aligned with respect to the soft islands. Most importantly, in the top region, where the concept of controlled cracking is applied, no uncontrolled cracks appear at strain levels below 35%. Instead, only an expansion of the controlled cracks is observed.

As mentioned, the critical strain for cracking can be tuned with the UV dose. Independently from the dose, however, a few observations illustrated by Figure 2 are generally valid. Firstly, controlled cracking always shows a lower critical strain than uncontrolled cracking. Otherwise, the concept of crack-control would not be useful in order to prevent statistical cracking. Secondly, at the regions with controlled cracks the critical strain for the formation of uncontrolled cracking is significantly increased. More precisely, uncontrolled cracking in addition to controlled cracking is generally observed at strain levels, where the average distance of uncontrolled cracks (90 µm shown in Figure 2c, bottom) falls below the minimum distance of controlled cracks, which is identical to the distance of the soft island (45 µm shown in Figure 2, top). This proves that the expansion of controlled cracks does release tensile strain in a very similar way as statistical (non-controlled) cracking does.

In summary, for the hardened PDMS surface the reported concept allows an increase of the critical strain for uncontrolled cracking. This maximum strain enabled with the concept is depending on the UV dose applied and the distance of the soft islands. In the example shown in Figure 2 the maximum strain is at about 35%. On the other hand, due to the relatively low UV dose, the hardened film itself does not crack at strains below 18%. In other words, the critical strain could only be increased by a factor of two. As functional thin films, like metal interconnects, crack at strains well below one percent, for these films a much larger increase of the critical strain will be required in order to enable stretchable electronics [31].

### 2.2. Cracking of the Silver Film on Top of the Hardened PDMS

In the next step it is investigated if the approach can be adopted to silver interconnects. The interconnects are deposited by electroless deposition. This method enables selective growth of the silver at the sites formerly hardened [21].

**Figure 3:**
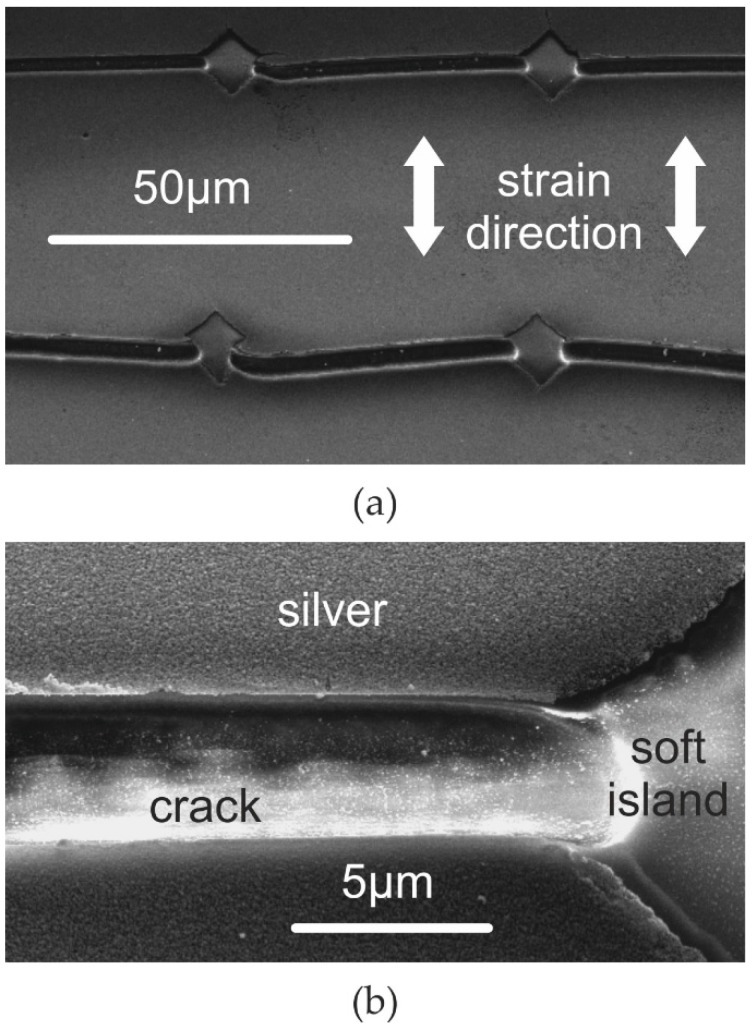
Scanning electron micrographs of PDMS surface with controlled cracks kept under tensile strain of 10% during silver deposition and at the same strain during electron microscopy: (**a**) four soft islands after controlled cracking; (**b**) a single controlled crack and the transition between crack and soft island (note, in contrast to Figure 2, the pattern shown here is coated with silver).

**Figure 4:**
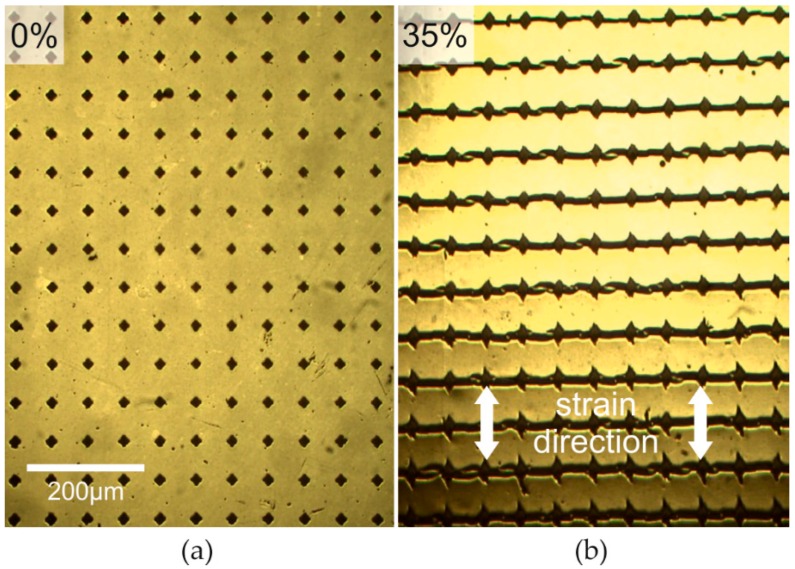
Optical micrographs of silver film deposited on the PDMS surface treated with UV (30 J·cm^−2^) including untreated soft islands (compare Figure 2a, top area, island distance: 45 µm): before (**a**), and after, mechanical stretching by 35% (**b**).

Therefore, the soft islands are not covered with silver. This is achieved without any further masking or alignment. In fact, even if the cracks are already opened no silver is deposited within the crack region, as this region was freshly opened during cracking. This self-alignment of the silver films at the crack- and island-free sites is shown in Figure 3. The silver films can also be deposited before creation of the controlled cracks. In that case, after the deposition only the soft island sites are free of silver (see Figure 4a).

**Figure 5:**
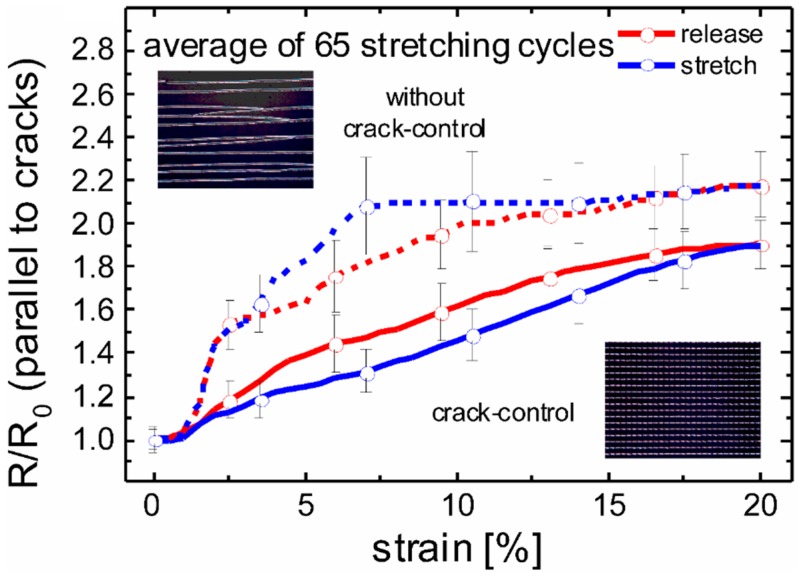
The change of the resistance in the direction parallel to the cracks during stretching perpendicular to the cracks; the two upper curves show the resistance change of a metal film without crack-control. The two lower curves show that crack-control results in a smaller resistance change under strain. The inset micrographs show the corresponding silver films after stretching. The length of the tested interconnects was about 7 mm.

During stretching the composite consisting of the hardened PDMS and the silver film cracks in a very similar way as it was observed for the hardened PDMS surface alone (Figure 2). Here, at mechanical strains as large as 35%, no additional uncontrolled cracking is observed (Figure 4b). This is remarkable because without crack-control the critical strain for cracking of smooth metal films is <1% [31,32]. This increase of the critical strain for statistical cracking by a factor >35 is significantly higher than the value of 2 found for hardened PDMS surfaces without silver coating. The electrical conductivity of the silver film strongly depends on the current direction. Obviously, in the direction perpendicular to the cracks the silver films are not conductive. However, in the other direction the silver lines are in parallel connection. The crack expansion does change the distance of the ribbons only, which leads to a slight change of the resistance (see Figure 5). Remarkably, this change is even smaller using crack-control as compared to a statistically cracked film, although the density of controlled cracks is higher at a given strain (see Figure 5, inset micrographs). This fact accounts for the more accurate alignment of controlled cracks.

**Figure 6:**
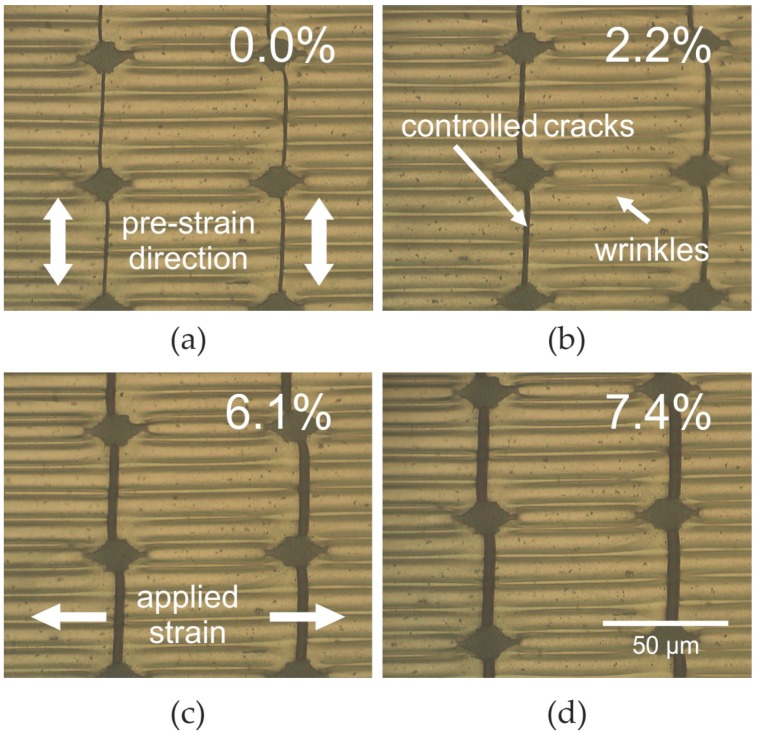
Optical micrographs of silver film containing controlled cracks and wrinkles under various strain of (**a**) 0%, (**b**) 2.2%, (**c**) 6.1%, and (**d**) 7.4%.

The drawback of the cracked interconnects shown in Figure 4 and Figure 5 is that they enable stretching in one direction only. Strain applied parallel to the cracks will lead to destruction of the conductive interconnect. As most applications for stretchable electronics require stretchability in any direction, a combination of controlled cracks and wrinkles appears promising. This is because wrinkled metal films on the one hand can be stretched in one direction and stay conductive in the same direction. On the other hand the wrinkles must be confined into narrow ribbons to prevent reorientation under changing strain direction [21]. The cracks can provide this confinement. Additionally, they enable stretching perpendicular to the wrinkles. Figure 6 shows the resulting interconnect layers that can be stretched in any direction without losing conductivity. For preparing such interconnects the substrate is first stretched in the pre-strain direction emphasized in Figure 6a. Under that pre-strain, the substrate is UV-exposed through the shadow mask for defining the hardened sites and the soft islands. Afterwards the silver film is deposited. At this stage the film is still flat. Wrinkles are formed by releasing the pre-strain (see Figure 6). The controlled cracks are formed by stretching the substrate perpendicular to the pre-strain (indicated in Figure 6c as applied strain). The pre-strain can either be released before or after stretching. The resulting pattern consist of wrinkled ribbons separated by cracks. It is stretchable in any direction. Stretching in the direction of the former pre-strain causes flattening of the wrinkles. During that flattening the sheet resistance of the ribbons is even slightly decreased by about 1% at 30% strain [21]. Importantly, stretching the ribbons perpendicular to the wrinkles leads to expansion of the controlled cracks only. This phenomenon is shown in Figure 6. With the applied strain being increased from 0% (Figure 6a) to 7.4% (Figure 6d) the crack width also increases. This crack expansion leads to an increase of the sheet resistance that is very similar to the behavior of wrinkle-free interconnects, which is shown in Figure 5. 

Being stretchable in two orthogonal directions at the same time means being stretchable in any planar direction that can always be considered a superposition of these two directions. The combination of controlled cracks and wrinkles enables arbitrary and changing stretching directions, as well as the application of wrinkling without the described restriction of a maximum surface coverage.

## 3. Discussion

In the field of elastically-stretchable electronic networks, cracking has so far been considered to be an undesired mechanism of destruction. However, the crack itself is—depending on the strain—a nanoscale to microscale feature that releases mechanical stress. The presented results show that cracking does not necessarily happen statistically. In fact, cracks can be started at a certain starting point, directed into a well-defined direction and, most importantly, stopped at a well-defined destination. Having reached that destination, the cracks are stabilized. That means that the crack tips do not propagate anymore, but the cracks can still open under strain.

In contrast to former reports, planar structures of very low surface coverage are applied in order to realize crack-control and enable large area stretchable metal interconnects. This approach for crack-control is very facile as it does not rely on photoresist, solvents, or vacuum processing. Hence, it is more compatible with soft materials than photolithography. Additionally, it enables smaller patterns, considering the cracks’ width at low strain. For the definition of the patterns only a chromium mask is put in direct contact with the stretchable substrate and illuminated by UV. The resulting hardened surface stays stretchable by about 18% (see Figure 2) which enables simple separation of the substrate from the mask without introducing cracks. The controlled cracks can then be introduced before or after electroless deposition of a silver film. In both cases the silver film is self-aligned and excludes the soft islands and the cracks. The cracking of the composite of hardened PDMS and silver film is controlled in a very similar way as observed for the pure hardened PDMS surface. The increase of the threshold for statistical cracking is much larger for the composite and further increases with the silver thickness, giving a hint that controlled cracking may be applied to thicker stacks.

Importantly, the designer of a stretchable electronic network based on the described interconnects can accurately predict where cracks are situated and what strain direction is released. Brittle devices can be placed close to the cracks, so external strain will not damage them. Former approaches avoided cracking in local islands [13,23]. However, a stretchable large-area connection technology remains a challenge [14]. The presented work focuses on that issue. It enables large area stretchable metal networks serving as interconnects for local rigid devices.

The resulting metal interconnects shown in Figure 3, Figure 4 and Figure 5 are stretchable in one direction. In principle a network based on many of such interconnects with different crack-direction could indeed be stretchable in arbitrary directions. However, in that case different strain directions for starting the cracks (compare Figure 2) would be needed, which would complicate the design of the network. Therefore, it appears promising to use networks with each interconnect being stretchable in any direction even for one strain direction only. Figure 6 shows such interconnects combining wrinkles and cracks. One stretching direction will cause flattening of the wrinkles, whereas the perpendicular direction opens the controlled cracks (this phenomenon is shown in Figure 6). In both cases the resistance is changed, reversibly rendering these interconnects stretchable in arbitrary directions.

Future work will focus on the realization of stretchable electronic circuits based on the interconnects presented in this paper. 

## 4. Materials and Methods

### 4.1. Sample Preparation

The PDMS substrates of around 1 mm thickness are prepared by curing a mixture (1:10) of curing agent and PDMS precursor (Sylgard 184, Dow Corning, Midland, MI, USA) on an indium tin oxide coated glass substrate for 2 h at 80 °C. The substrate is then illuminated by an excimer lamp (Xeradex, Osram, Munich, Germany) (10 min, λ = 172 nm, *I* = 45 mW·cm^−2^, lamp to substrate distance: 8 mm) through a chromium mask. It has to be noted that after absorption of the UV in atmosphere (8 mm) and mask, only a fraction of the lamp dose reaches the PDMS surface. This exact dose has not been determined. The mask was prepared by lithographic patterning of a 300 nm thick chromium film on sapphire. 

### 4.2. Electroless Deposition

The electroless deposition (ELD) solution 10A (see [21]) is put in a beaker and the PDMS substrate is placed on top of the solution with the UV exposed surface facing downwards. This way the substrate is swimming on top of the solution which prevents agglomerates formed in the solution from falling on the sample surface.

### 4.3. Stretching Experiments

The stretching experiments are conducted using a self-made stretching apparatus that compensates the Poisson effect. Resistance was measured during stretching using a Keithley 199 System DMM/Scanner (Keithley Instruments, Inc., Cleveland, OH, USA).

## Abbreviations

The following abbreviations are used in this manuscript:

PDMS	polydimethylsiloxane
UV	ultraviolet
ELD	electroless deposition
